# COVID-19 in Liver Transplant Recipients: A Systematic Review

**DOI:** 10.3390/jcm10174015

**Published:** 2021-09-05

**Authors:** Chiara Becchetti, Sarah Gabriela Gschwend, Jean-François Dufour, Vanessa Banz

**Affiliations:** 1 University Clinic for Visceral Surgery and Medicine, Inselspital, Bern University Hospital, University of Bern, 3010 Bern, Switzerland; sarah.gschwend@students.unibe.ch (S.G.G.); jf.dufour@lasource.ch (J.-F.D.); Vanessa.BanzWuethrich@insel.ch (V.B.); 2 Hepatology, Department of Biomedical Research, University of Bern, 3010 Bern, Switzerland

**Keywords:** solid organ transplantation, liver injury, immunosuppressant, SARS-CoV-2, humoral response, vaccination

## Abstract

Liver transplant (LT) recipients are considered a vulnerable population amidst the COVID-19 pandemic. To date, available data have been heterogeneous and scarce. Therefore, we conducted a systematic literature review identifying English-language articles published in PubMed between November 2019 and 30 May 2021. We aimed to explore three areas: (1) outcome and clinical course; (2) immunological response after COVID-19 in LT recipients; and (3) vaccination response. After systematic selection, 35, 4, and 5 articles, respectively, were considered suitable for each area of analysis. Despite the heterogeneity of the reports included in this study, we found that gastrointestinal symptoms were common in LT recipients. The outcome of the LT population was not per se worse compared to the general population, although careful management of immunosuppressive therapy is required. While a complete therapy discontinuation is not encouraged, caution needs to be taken with use of mycophenolate mofetil (MMF), favoring tacrolimus (TAC) use. Although data conflicted about acquired immunity after SARS-CoV-2 infection, vaccine immunogenicity appeared to be low, suggesting that the level of surveillance should be kept high in this population.

## 1. Introduction

Severe acute respiratory syndrome coronavirus 2 (SARS-CoV-2) has first been identified in Wuhan city, Hubei Province, China as the pathogen responsible for several cases of severe pneumonia during November 2019, subsequently defined by the World Health Organization (WHO) as Coronavirus disease 2019 (COVID-19). Typical symptoms of COVID-19 include fever, cough, dyspnea, fatigue, myalgia, gastrointestinal manifestations, and impairment of smell and/or taste [1,2,3]. The course of the disease ranges from asymptomatic or mild [4] to severe manifestations, mainly with respiratory features, leading to respiratory insufficiency, acute respiratory distress syndrome (ARDS), and in some cases to death. Age, male gender, and comorbidities have been established as risk factors for a more severe course of the disease and for mortality [5,6].

Since March 2020, COVID-19 has spread worldwide, has been declared a pandemic by the WHO, and has rapidly become a public health matter with several unmet issues. As of 16 July 2021, there were over 188 million confirmed cases and over 4 million reported deaths worldwide [7].

While knowledge on disease evolution, risk factors, clinical manifestations, and optimal management of affected individuals is progressively increasing, treatment guidelines are difficult to standardize when taking into account specific categories of patients. In this regard, solid organ transplant (SOT) recipients, and among them, liver transplant (LT) patients, may represent a potentially high-risk population. Concerns have been raised regarding immunosuppression therapy, including SARS-CoV-2-associated liver injury [8] and a possible impairment of the immunological response.

In December 2020, encouraging results on the safety and efficacy profile of the first anti-SARS-CoV-2 vaccines were published [9,10], paving the way for a large-scale vaccination campaign. However, most special populations were excluded from the pivotal studies of these vaccines, and therefore, real-life observations on efficacy and safety are necessary.

Data regarding the management of immunosuppression therapy in LT recipients affected by COVID-19, as well as information on the course of the disease, outcome, and immunological response both to the infection and vaccination, remain scarce.

The aim of this review was therefore to analyze and summarize the published literature concerning LT recipients with COVID-19.

## 2. Materials and Methods

A systematic literature review was conducted identifying PubMed English-language articles published between November 2019 and 30 May 2021.

We structured our search on three areas, using different MeSH terms. First, we aimed to analyze outcome and clinical course in LT recipients; second, we aimed to analyze immunological response after COVID-19 in LT recipients; and third, we aimed to analyze vaccination response.

For the first purpose, the MeSH terms used were ‘‘COVID-19” (and related terms: 2019 novel coronavirus, SARS-CoV-2 infection, 2019-nCoV infection) AND ‘‘liver transplant” (and related terms: orthotopic liver transplant (OLT), hepatic transplant, liver transplantation, solid organ transplant).

For the second purpose, the MeSH terms used were ‘‘COVID-19” (and related terms) AND “liver transplant” (and related terms) AND “humoral response” (and related terms: serology, immune response, T-cell response).

For the third purpose, the MeSH terms used were ‘‘liver transplant” (and related terms) AND “COVID-19 vaccines”.

Original articles, case reports, case series, commentaries, letters to the editor, and review articles were considered. Additional articles were considered on the basis of the reference lists of the included studies. Two reviewers independently evaluated titles and abstracts for inclusion. Only well-characterized adult transplant recipients were included. Articles with known duplications were excluded. When feasible, information on LT recipients summarized in mixed cohorts of SOT patients were extracted and analyzed. Systematic selection was performed according to the Preferred Reporting Items for Systematic Reviews and Meta-Analyses (PRISMA) [11]. Data extraction was conducted independently by two researchers (SGG and CB), using the text, tables, and figures of the original published articles. Independently, the overall quality was also evaluated and graded according to the Newcastle–Ottawa scale (NOS) for assessing the quality of the observational studies and converting the results to the Agency for Health Care Research and Quality (AHRQ) standards (good, fair, and poor) where applicable. When disagreement was present, an open discussion led to a final consensus. All the reported patients’ demographic and clinical characteristics, baseline immunosuppressant medications and modifications during the course of the infection, need for intensive care unit (ICU) and/or mechanical ventilation (MV), and outcome were collected. A meta-analysis to investigate association between baseline characteristics, immunosuppression, and outcomes was not performed because of the lack of sufficient data and the high heterogeneity between the different studies. For the second and third aims, we collected the data regarding the type of assay used to assess immunity and the type of vaccines applied. The principal measures used were the median, mean, standard deviation, and incidence as pooled results.

## 3. Results

### 3.1. Study Selection

**Figure 1:**
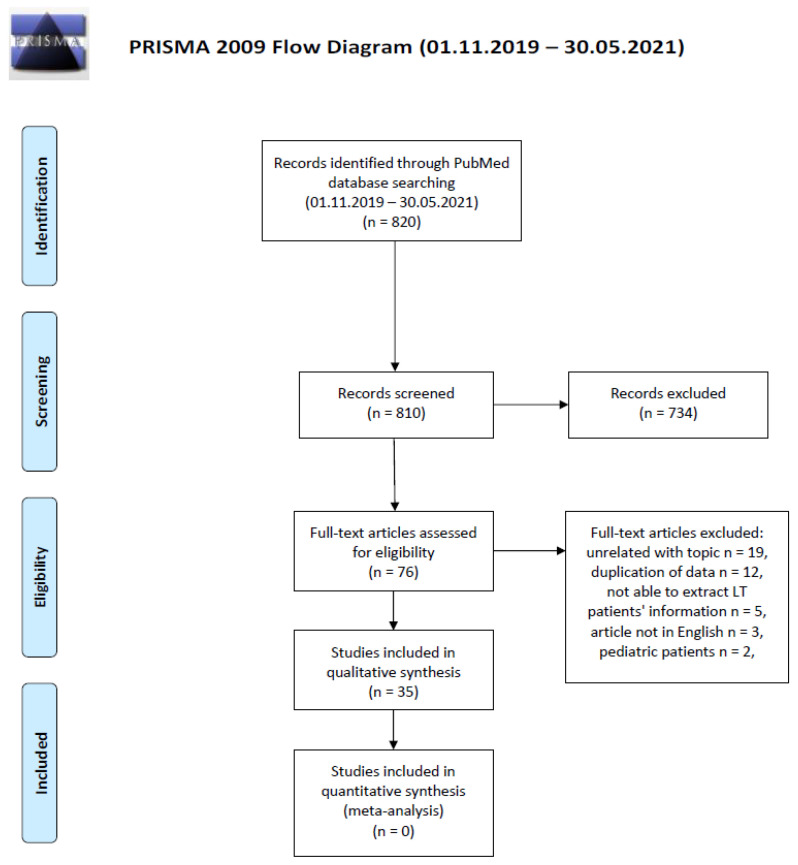
Flow diagram of the systematic literature search according to the PRISMA statement.

For the first aim, 820 papers met the research criteria applied, of which 76 articles were considered suitable for evaluation. Preliminary reports subsequently published as extended analyses were considered duplications and therefore not included in the final analysis. In addition, data duplication for survey-based studies could not be completely ruled out. Therefore, we restricted our final selection to 35 articles, including a total of 1076 patients. No randomized control trials were found, and only two studies were prospectively designed; the remaining 33 articles were retrospective studies, case reports or case series, editorials, or letters to the editor. Five studies reached “good quality” according to the NOS converted in the AHRQ standards. One study reached “fair quality”, and the other studies were rated as “poor quality”. The selection process followed the PRISMA guidelines and is summarized in Figure 1.

For the second and the third aims, 18 and 19 papers met the research criteria applied, respectively. Of these, four and five articles respectively were considered suitable for evaluation. No randomized control trials were found. Only two studies were prospectively designed; the remaining articles were retrospective studies, case reports or case series, editorials, or letters to the editor.

### 3.2. Study Population Characteristics, Clinical Course, and Management of Immunosuppression

Overall, 1076 patients were pooled. Mean age was 54.5 ± 12.1 years, with male gender being prevalent (*n* = 553, 66.8%). Extensive information on comorbidities was available in 30 papers. Diabetes mellitus type 2, arterial hypertension, and obesity were present in 38.6%, 43.5%, and 16.0% of patients, respectively. A history of previous neoplasia was described in three reports, identifying 23 out of 832 patients (2.8%). In the majority of patients, infection with SARS-CoV-2 occurred 79.7 months after LT. The demographic characteristics and main outcomes are summarized in Table 1.

Regarding the incidence of COVID-19 infections in LT recipients, only the SETH cohort provided data, showing that the incidence of COVID-19 in liver transplant recipients compared to the general population (837.41 cases/ 105 patients vs. 311.93 cases/ 105 people) was almost double [17].

On the other hand, the COVID-LT cohort recorded 57 confirmed SARS-CoV-2 infections out of 11,790 patients in regular follow-ups, resulting in an incidence of 483.46 cases/ 105 patients [13]. Another report from Germany documented, using either serology or PCR-swab test, present or past SARS-CoV-2 infection in 3.7% of their LT recipients during the study period (May and August 2020) [41].

The most frequently described clinical presentation was fever (61.4%), followed by cough (58.6%) and dyspnea (36.2%). Webb et al. [45] reported general “respiratory symptoms”, which were experienced by the 77% of the LT recipients included in the study. Gastrointestinal symptoms including vomiting, diarrhea, nausea, and abdominal pain were strongly represented (159/569 patients, 27.9%). In the aforementioned study, the proportion of patients with gastrointestinal symptoms was higher among LT recipients compared to the nontransplant cohort (30% vs. 12%, *p* < 0.0001), whereas no significant difference was observed with respect to respiratory symptoms. On the same line, Belli et al. [14] found diarrhea as the presenting symptom in 55 LT recipients, corresponding to 22.6%.

Concerning immunosuppression therapy, data on basal immunosuppression (IS) therapy and on subsequent management during the course of infection was available for 33 and 29 studies, respectively. The data are summarized in Table 2.

In the study by Colmenero et al. [17] patients receiving MMF or in whom an attempt was made to completely withdraw immunosuppression were more prevalent in the severe COVID-19 group (*p* = 0.014, and *p* = 0.016 respectively). Conversely, tacrolimus-based immunosuppression was more frequent in the nonsevere COVID-19 group, albeit without statistical significance (*p* = 0.113). Similar findings regarding calcineurin inhibitor (CNIs)-based regimens were observed in the COVID-LT study, where the continuation of CNIs therapy after COVID-19 diagnosis was higher among survivors (64% vs. 42.8%) [47]. Indeed, in the study of Belli et al. [14], after multivariable analysis, the use of TAC was confirmed to be independently associated with a reduced mortality risk (HR, 0.55; 95% CI, 0.31–0.99). Additionally, in the Spanish cohort, survival curves illustrated the negative prognostic impact of MMF, particularly at doses higher than 1000 mg/day. In agreement with this finding, in patients receiving full-dose of MMF at baseline (i.e., 2000 mg/day), complete drug withdrawal showed a trend towards reduced severe COVID-19 (41.7% vs. 69.2%, *p* = 0.16) [17].

Overall, 375 out of 1064 (35.2%) patients were managed in an outpatient setting, whereas 64.8% were hospitalized. Of the hospitalized patients, 158/689 (22.9%) were admitted to an ICU. Death was reported in 135 cases. In the COVID-LT study, case fatality was estimated at 12% (95% CI 5–24%), which increased to 17% (95% CI 7–32%) among hospitalized patients [13], whereas Rabiee and coauthors found a 22.3% case fatality rate [40]. In the study by Webb and coauthors [45], case fatality was 19% (vs. the 27% reported for the comparison cohort, *p* = 0.046), with the propensity-score-matched analysis showing that LT did not significantly increase the risk of death in patients with SARS-CoV-2 infection (absolute risk difference 1.4% (95% CI 7.7–10.4)). Colmenero et al. [17] described a mortality rate of 18% among LT patients.

In Webb et al., multivariate analyses showed that factors significantly associated with death were: increased age (OR 1.06 (95% CI 1.01–1.11) per 1 year increase, *p* = 0.031), presence of nonliver cancer (OR 18.30 (1.96–170.75); *p* = 0.011), and higher baseline serum creatinine (OR 1.57 (1.05–2.36) per 1 mg/dL increase) *p* = 0.028) [45]. Results derived from the multivariate analysis performed within the SETH cohort study identified the following independent predictors: Charlson comorbidity index (relative risk (RR) = 1.28 (95% CI 1.05–1.56), male gender (RR = 2.49; 95% CI 1.14–5.41), dyspnea at diagnosis (RR = 7.25; 95% CI 2.95–17.82), and baseline immunosuppression containing MMF (RR = 3.94; 95% CI 1.59–9.74) [17]. Belli et al. reported risk factors associated with worse prognosis including advanced age (>70 vs. <60 years, HR 4.16; 95% CI 1.78–9.73) and the use of TAC [14].

Despite theoretically higher levels of immunosuppression, only the report by Belli et al. [14] mentioned time since LT as an independent factor associated with poor outcome in univariate analysis. On the other hand, Colmenero et al. [17] showed that the time from LT had no impact on the risk of suffering from severe COVID, a finding that was confirmed by Webb et al. [45], who reported no association between death and time since LT.

Lastly, Rabiee et al. showed that the incidence of acute liver injury (defined by ALT 2-5x ULN) was not higher in LT recipients when compared to age- and gender-matched nontransplant patients with chronic liver disease and COVID-19 (47.5% vs. 34.6%; *p* = 0.037). The presence of liver injury during COVID-19 in LT recipients was significantly associated with mortality (OR 6.91 (95% CI: 1.68–28.48), *p* = 0.007) and ICU admission (OR 7.93 (95% CI: 1.75–35.69), *p* = 0.007) [40]. In the US study of Hadi et al., considering only LT recipients, only 18 patients (7.5%) experienced the composite outcome including mechanical ventilation and death at 30 days. This rate was lower when compared to that for recipients of other organ transplants [23].

### 3.3. Immunological Response after COVID-19 in LT Recipients

Regarding the immunological response after SARS-CoV-2 infection in LT patients, only four studies were considered, including a total of 91 LT recipients. However, all of these studies examined different types of tests/assays directed toward different targets, and data could not always be extrapolated to LT recipients alone, as shown in Table 3, making a pooled analysis not feasible.

Caballero-Marcos et al. [48] showed a decline over time of IgG-antinucleocapsid, with lower incidence at 3 months (77.4% vs. 100%, *p* < 0.001) and at 6 months (63.4% vs. 90.1%, *p* < 0.001) when compared with a matched cohort of immunocompetent subjects. A more comprehensive analysis performed in 28 SOT patients (of which five were LT patients) of the immunological response, which also considered T-cell responses, showed that the overall response was not impaired in the SOT patients. However, when the humoral response was considered alone, there was some delay in mounting a response compared to the immunocompetent control group [49].

### 3.4. COVID-19 Vaccine Immunogenicity in LT Recipients

Regarding response to COVID-19 vaccines, we considered five studies. Overall, the studies included 269 LT recipients (Table 4). However, analogously to those regarding the immunological response after COVID-19, the included studies considered different types of tests/assays, and data could not always be extrapolated to LT recipients alone, making comparisons difficult. Two studies evaluated side effects after receiving one dose of mRNA-1273 or BNT162b2 vaccine or two doses of BNT162b2 vaccine. Both studies showed local and systemic side effects in proportions comparable to those in pivotal studies for RNA vaccines.

Concerning immunogenicity, four studies, although using different assays, evaluated the humoral response recording seroconversion rates between 29 and 50% [52,53]. Two studies emphasized that the use of antimetabolites as immunosuppression was a risk factor for reduced serum conversion rates [53,54]. Only one study also analyzed T-cell response, which was described as reduced [55], and only one of the included studies considered only LT recipients [53].

## 4. Discussion

At the beginning of the COVID-19 pandemic, SOT recipients, including LT recipients, were considered a vulnerable population, raising the question as to whether they would be at particular risk for severe disease and graft injury given their immunocompromised state and high prevalence of metabolic comorbidities. The aim of our study was to systematically pool all the available literature on this topic. We found that middle-aged men with metabolic comorbidities were the main target for the infection. Even though respiratory problems represented the main clinical feature in LT patients, a high percentage of gastrointestinal symptoms were also reported. Approximately 70% of LT patients with SARS-CoV-2 infection were hospitalized. Modification/reduction of IS was common, particularly for MMF, although complete withdrawal of all IS was rarely observed. With respect to outcome, a case fatality rate ranging between 12 and 22% was described in the major reports accessible for this analysis. Interestingly, when compared to the control population, outcomes were not worse in the LT recipient group.

There seems to be a difference in the immune response to the SARS-CoV-2 infection and the immune response as acquired by vaccination. In the first case, the data conflict, but if we consider the response mediating the neutralizing activity (anti-spike protein IgG and T-cell mediated), it seems that there is a similar response, albeit probably slightly delayed, in LT recipients compared to immunocompetent subjects. On the other hand, vaccine-induced immunogenicity seems to be defective.

Although a considerable number of patients were included in the present study, the quality of the manuscripts analyzed makes it difficult to consolidate associations and predictive factors regarding COVID-19 and the LT population.

The epidemiological distribution of the disease is superimposable to that of the general population [2,57], with COVID-19 being mainly prevalent in middle-aged males.

Recently, new findings have highlighted how obesity [58], diabetes type II [59], and arterial hypertension [60] are associated with a more severe course of COVID-19 and hence a poorer outcome. Despite the high prevalence of these metabolic conditions in LT recipients [61,62] and more specifically in the present cohort, this did not seem to negatively affect the prognosis of the current study population.

Interestingly, the presence of gastrointestinal complaints (28%) was considerably higher among LT recipients. A recent review on gastrointestinal symptoms in COVID-19 [63] showed a high heterogeneity in incidence (ranging between 3 and 79%), with other large cohorts of patients reporting rates of gastrointestinal symptoms between 5 and 15% [60,64]. It is widely accepted that SARS-CoV-2 enters host mucosal cells via the cell receptor angiotensin-converting enzyme-2 (ACE-2) and the transmembrane serine protease 2 (TMPRSS2), which are also highly expressed in the absorptive enterocytes from the ileum and colon [65]. Once the virus enters the enterocytes, it can start replication and its cytopathic effect [66]. The gut microbiome can be significantly altered by SARS-CoV-2 through several mechanisms (e.g., proinflammatory cytokines, perturbation in the gut–lung axis, medications, changing ratio of pathogenic organisms) leading to clinical manifestations such as diarrhea and vomiting [67]. LT patients are also known to have an extremely vulnerable gut microbiomes [68,69], and immunosuppressive fluctuation in trough level can interfere with gut flora stability [70]. Furthermore, it was observed that patients with digestive symptoms, probably not recognized from the outset as symptoms associated with COVID-19, had a significantly longer time from onset to admission than patients without digestive symptoms (9.0 days vs. 7.3 days, *p* = 0.013) [71]. Interestingly, a recent study aiming to analyze the gut inflammatory response in immunocompetent subjects infected with SARS-CoV-2 highlighted the absence of a proinflammatory response in the gastrointestinal tract despite detection of SARS-CoV-2. Additionally, this study showed reduced mortality in patients with COVID-19 presenting with GI symptoms. Therefore, the authors speculated on a potential role of the gastrointestinal tract in attenuating SARS-CoV-2-associated inflammation [72].

In the current cohort, the mortality rate and case-fatality rate did not seem to exceed those expected in the general population. Indeed, the hypothesis that LT is a possible associated factor for a pejorative outcome could not be confirmed. However, of note was the high rate of hospitalization, with 64.8% of LT recipients being admitted to a ward and 22.9% of such patients requiring intensive care. This may of course reflect a certain selection bias, with more LT recipients being hospitalized per se. Additionally, one must keep in mind that during the first wave, there were many logistical difficulties, which probably led to an underdiagnosis of asymptomatic or paucisymptomatic cases [73].

With regard to the management of immunosuppression, not all the information can be extrapolated, as most studies have had descriptive designs. The different nature of the immunosuppressive regimens adopted, often multiple, and the modification of these regimens during the course of infection make it difficult to provide any clear guidelines to this respect. However, it seems that a complete discontinuation of IS therapy was very rare and limited to extremely severe cases. Unfortunately, complete cessation of IS was not associated with improved prognosis [17] and should therefore only be considered as a last resort in selected cases.

In the Spanish cohort, patients receiving MMF were more prevalent in the severe COVID-19 group. Baseline immunosuppression containing MMF was identified as an independent predictor of mortality, whereas the withdrawal of IS was not. However, data on modifications in immunosuppressive therapy during the infection were not extensively available in this study [17] and reduction or discontinuation of MMF was recommended by the guidelines shared by experts at the beginning of the pandemic [74]. Therefore, whether the impact on outcome is attributable to the MMF itself or to its reduction/discontinuation remains objects of further investigation. In a preclinical setting, MMF showed promising results against Middle East respiratory syndrome (MERS); however, in vivo studies suggested that its use is likely to cause more harm than benefit against coronavirus (CoV) infection [75]. Bearing in mind that MMF acts on activated lymphocytes with a cytostatic effect [76] and that SARS-CoV-2 has a cytotoxic effect on the same target [77], these synergistic effects may represent an additional risk factor and worsen the prognosis of LT patients taking MMF.

In Belli et al. [14], TAC was found to protect against worse outcomes in COVID-19 LT recipients. In vitro experiments have shown that TAC, and CNIs in general, are capable of inhibiting human CoV growth, mainly by acting on the cyclophilin pathway [78]. Additionally, by modulating T-cell activation, CNIs may act on reducing the deleterious effect of the COVID-19 late inflammatory phase [79].

Concerning the immunological response to SARS-CoV-2 infection, contrasting results were seen. However, most of the studies considered analyzed only the humoral response and in particular used an assay, the antinucleocapsid test, more suitable to evaluate preexisting exposure to the virus than to assess protective efficacy. Indeed, these antibodies have low or no neutralizing activity [80]. On the other hand, the analysis of the T-cell-mediated response in LT recipients showed similar results compared to that for immunocompetent subjects. Further studies are therefore necessary that take into account the complexity of the immune response in vivo and of the interplay among native, humoral, and T-cell immunity.

In contrast, all studies evaluating vaccine immunity have demonstrated reduced immunogenicity of LT recipients, and SOT recipients in general. It is possible, as pointed out by Mazzola et al. [52], that this reduced response is more evident in other SOT recipients, such as those for kidney or heart SOTs, than in LT recipients. In this line, the serum conversion data of the only cohort that included only LT recipients were actually higher than the average of the other studies, which included mixed cohorts. A deterrent role could be played again by more sustained immunosuppression (with dual or triple regimens) and by the use of antimetabolites [53]. Reduced immunogenicity in LT recipients has already been demonstrated with other respiratory virus (e.g., influenza) vaccines [81], suggesting that more than the standard dose may be needed to achieve protective immunogenicity [82].

**Figure 2:**
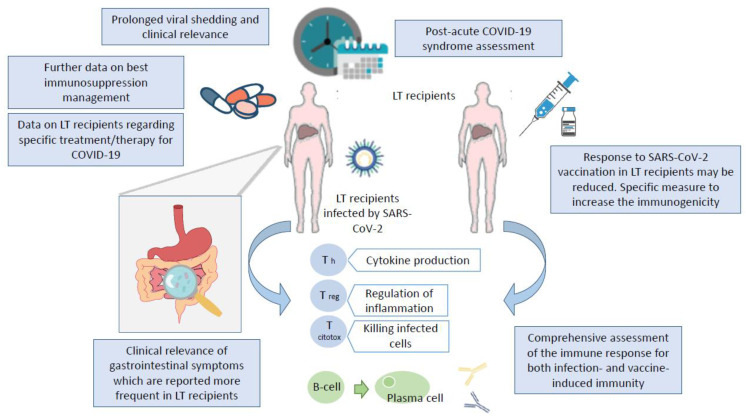
Graphical representation of the key unresolved issues for SARS-CoV-2 infection in LT recipients.

Several limitations affected the current study, mainly because of the high heterogeneity and quality of the majority of the studies considered, leading in several cases to incomplete information. Therefore, more than a few research questions remain open and will need future investigation (Figure 2).

## 5. Conclusions

Despite the heterogeneity of the reports included in this study, we are able to show that the middle-aged man with metabolic comorbidities represents the main target of COVID-19 among LT recipients, as is the case in nontransplanted patients. Gastrointestinal symptoms are very common in LT recipients with COVID-19, and particular attention should be paid to these complaints as a surrogate marker for COVID-19, even in the absence of fever or respiratory problems. The outcome of the LT population is nevertheless similar to that of the general population. This is, in part, also likely due to a more cautious management of immunosuppressive therapy, paying particular attention to the use of MMF and TAC while discouraging the complete discontinuation of all immunosuppression. LT recipients should be vaccinated, and great attention to protective measures should be maintained in these individuals even after a regular course of vaccination.

## Figures and Tables

**Table 1:** General characteristics of the included studies.

First Author	Country	Number of Patients (m/f)	Mean/Median Age	Comorbidities, *n* (%)	Outcome
Alconchel et al. [12]	Spain	3 (1/2)	64 (61–68)	Hypertension 2 (67)	CFR 67% ICU 100% ARDS 33%
				Diabetes 1 (33)	
				Stroke 1 (33)	
				Hypothyroidism 1 (33)	
Becchetti et al. [13]	Europe	17 (12/5)	Median 61 (IQR 26)	Hypertension 8 (47)	CFR 24% ICU 12% ARDS 18%
				Diabetes 9 (53)	
				Cardiovascular disease 8 (47)	
				CKD 7 (41)	
				Obesity 5 (29)	
				Respiratory disease 2 (12)	
				Cancer 8 (47)	
Belli et al. [14]	Europe	243 (171/72)	63 (55–69)	Hypertension 111 (46)	CFR 20% ICU 15% ARDS NA
				Diabetes 94 (39)	
				Cardiovascular disease 17 (7)	
				CKD 49 (20)	
				Obesity 46 (19)	
				Respiratory disease 25 (10)	
				Other 43 (18)	
Bösch et al. [15]	Germany	SOT 7; of them, 2 LT (1/1)	42 (18–65)	Hypertension 2 (100) CKD 1 (50)	CFR 0% ICU 0% ARDS NA
Choudhury et al. [16]	India	6 (6/-)	46 (38–52)	Hypertension 3 (50)	CFR 0% ICU 17% ARDS NA
				Diabetes 3 (50)	
				CKD 1 (17)	
				Hypothyroidism 1 (17)	
Colmenero et al. [17]	Spain	111 (79/32)	Median 65 (IQR 11)	Hypertension 64 (58)	Mortality rate 18% ICU 11% ARDS NA
				Diabetes 53 (48)	
				Cardiovascular disease 22 (20)	
				Respiratory disease 13 (12)	
Dale et al. [18]	USA	2 (-/2)	62 (58–65)	Cardiovascular disease 1 (50)	CFR 0% ICU 100% ARDS NA
				Respiratory disease 1 (50)	
				Rheumatoid arthritis 1 (50)	
				Psoriatic arthritis 1 (50)	
Dhampalwar et al. [19]	India	12 (11/1)	54 (45–63)	Diabetes 9 (75)	CFR 8% ICU NA ARDS NA
				Hypertension 4 (33)	
				Metabolic syndrome 1 (8)	
				Chronic rejection 1 (8)	
Eslami et al. [20]	Iran	1 (m)	69	CKD	CFR 100% ICU 100% ARDS NA
Felldin et al. [21]	Sweden	SOT 53; of them 8 LT and 3 kidney–LT (4/7)	60 (27–72)	Hypertension 1 (9)	CFR 9% ICU NA ARDS NA
				Diabetes 6 (55)	
				Cardiovascular disease 1 (9)	
				CKD 2 (18)	
				Obesity 2 (18)	
				Respiratory disease 2 (18)	
				Hypothyroidism 1 (9)	
				Sarcoidosis 1 (9)	
				Polymyalgia rheumatica 1 (9)	
				Psychosis 1 (9)	
				Cancer 1 (9)	
Fung et al. [22]	USA	SOT 10; of them, 1 LT (f)	80	Hypertension	CFR 0% ICU No ARDS No
				Diabetes	
				Cardiovascular disease	
				CKD	
				Respiratory disease	
				Hypothyroidism	
				Dementia	
Hadi et al. [23]	USA	2307 SOT; of them, 240 LT (NA)	NA for LT	**LT:**	**LT:** CFR NA ICU 10% ARDS NA
		Control group 2289 (1399/890)	Control group 55	NA	
				**Control group:**	
				Hypertension 2213 (92)	**Control group:** CFR 6% ICU 9% ARDS NA
				Diabetes 1420 (62)	
				Cardiovascular disease 1020 (45)	
				Respiratory disease 633 (28)	
Hann et al. [24]	UK	3 (2/1)	61 (47–69)	Hypertension 2 (67)	CFR 33% ICU NA ARDS NA
				Diabetes 3 (100)	
				CKD 2 (67)	
				Obesity 1 (33)	
				Other 1 (33)	
Hatami et al. [25]	Iran	1 (f)	30	Hypothyroidism	CFR 0% ICU NA ARDS NA
Hayashi et al. [26]	Japan	1 (m)	20	NA	CFR 0% ICU No ARDS No
Huang et al. [27]	China	1 (m)	59	HBV	CFR 100% MV Yes ARDS NA
Jamir et al. [28]	India	1 (m)	49	Obesity	CFR 0% ICU 100% ARDS NA
Kates et al. [29]	USA	SOT 4; of them, 1 LT (m)	67	Cirrhosis of allograft 1 (100)	CFR 0% ICU Yes ARDS NA
Kolonko et al. [30]	Poland	SOT 4; of them, 1 LT (m)	53	Ulcerative colitis 1 (100)	CFR 0% ICU No ARDS NA
Liu et al. [31]	China	1 (m)	50	NA	CFR 0% ICU 0% ARDS NA
Mansoor et al. [32]	USA	LT 125 (82/43) Control group 125 (85/40)	LT 57 (SD +/−19) Control group 60 (SD +/−15)	**LT:**	**LT:** CFR <8% ICU <8% ARDS NA
				Hypertension 29 (23)	
				Diabetes 20 (16)	
				CKD 24 (19)	
				Respiratory disease <10 (<8)	
				Heart failure <10 (<8)	**Control group:** CFR <8% ICU 9% ARDS NA
				(<8)	
				Ischemic heart disease <10 (<8)	
				**Control group:**	
				Hypertension 23 (18)	
				Diabetes 20 (16)	
				CKD 26 (21)	
				Respiratory disease <10 (<8)	
				Heart failure <10 (<8)	
				Ischemic heart disease <10 (<8)	
Mathiasen et al. [33]	Denmark	1 (f)	58	Hypertension	CFR 0% ICU 0% ARDS NA
				Obesity	
				Hyperlipidemia	
Mehta et al. [34]	USA	SOT 11; of them, 3 LT (2/1)	65 (62–68)	Hypertension 2 (67)	CFR 0% ICU 0% ARDS NA
				Cardiovascular disease 1 (33)	
				Diabetes 1 (33)	
				Obesity 2 (67)	
				HIV 3 (100)	
				HCV 3 (100)	
Modi et al. [35]	USA	1 (m)	32	HIV	CFR 0% ICU 0% ARDS NA
Niknam et al. [36]	Iran	2 (1/1)	53 (46–60)	Diabetes 1 (50)	CFR 0% ICU 0% ARDS NA
Nikoupour et al. [37]	Iran	1 (m)	35	Ulcerative colitis 1 (100)	CFR 0% ICU 0% ARDS NA
Prieto et al. [38]	Spain	1 (m)	52	Cardiovascular disease 1 (100)	CFR 0% ICU No ARDS NA
Qin et al. [39]	China	1 (m)	37	NA	CFR 0% ICU NA ARDS NA
Rabiee et al. [40]	USA	112 (61/51)	Median 61 (IQR 20)	Hypertension 59 (53)	CFR 22% ICU 27% ARDS NA
				Diabetes 51 (46)	
				Cardiovascular disease 15 (13)	
				Obesity 26 (23)	
				Respiratory disease 18 (16)	
				Metabolic syndrome 22 (20)	
				Cancer 7 (6)	
				HIV 1 (1)	
Rauber et al. [41]	Germany	8 (NA)	57 (26–70)	Hypertension 5 (63)	CFR 0% ICU 0% ARDS NA
				Diabetes 2 (25)	
				Cardiovascular disease 1 (13)	
				CKD 4 (50)	
				Obesity 2 (25)	
Sessa et al. [42]	France	1 (m)	58	Hypertension	CFR 0% ICU 0% ARDS NA
Terrabuio et al. [43]	Brazil	4 (1/3)	52 (42–62)	Hypertension 1 (25)	CFR 0% ICU 0% ARDS NA
				Diabetes 1 (25)	
				Obesity 2 (50)	
				Chronic rejection 1 (25)	
				Crohn’s disease 1 (25)	
Waisberg et al. [44]	Brazil	5 (4/1)	60 (34–69)	Hypertension 3 (60)	CFR 40% ICU NA ARDS NA
				Diabetes 1 (20)	
				Cardiovascular disease 1 (20)	
				Obesity 2 (40)	
				Respiratory disease 1 (20)	
Webb et al. [45]	International	LT 151 (102/49) Control group 627 (329/298)	LT Median 60 (IQR 47–66) Control group 73 (IQR 55–84)	**LT:**	**LT:** CFR 19% ICU 28% ARDS NA
				Hypertension 63 (42)	
				Diabetes 65 (43)	
				Cardiovascular disease 22 (15)	
				Obesity 44 (29)	
				Respiratory disease 8 (5)	**Control group:** CFR 27% ICU 8% ARDS NA
				Cancer 8 (5)	
				**Control group**:	
				Hypertension 241 (38)	
				Diabetes 144 (23)	
				Cardiovascular disease 202 (32)	
				Obesity 158 (25)	
				Respiratory disease 160 (26)	
				Cancer 92 (15)	
				Stroke 73 (12)	
Wei et al. [46]	China	1 (m)	61	NA	CFR 0% ICU NA ARDS NA

Abbreviations: ICU: intensive care unit; ARDS: acute respiratory distress syndrome; HBV: hepatitis B virus; HIV: human immunodeficiency virus; HCV: hepatitis C virus; SOT: solid organ transplantation; LT: liver transplant; NA: not applicable; CKD: chronic kidney insufficiency; IQR: interquartile range; CFR: case fatality rate.

**Table 2:** Information regarding immunosuppressant regimen and its modification in during COVID-19.

First Author	Number of Patients	Baseline IS, *n* (%)	Modification IS	Type of Modification
Alconchel et al. [12]	3	TAC + steroid, 1 (33) TAC + MMF + steroid, 2 (67)	Yes	TAC reduction 33% MMF withdrawal and TAC reduction 33% MMF withdrawal 33%
Becchetti et al. [13]	17	**Single agent** Cys, 1 (6) TAC, 4 (24) Everolimus, 1 (6) MMF, 2 (12) **Combination** CNIs + MMF, 7 (41) CNIs + steroids, 2 (12)	Yes	Reduction 29% Withdrawal 18%
Belli et al. [14]	243	**Single agent** Cys, 13 (5) Cys + MMF, 9 (4) Cys + steroid, 3 (1) Cys + MMF + steroid, 4 (2) TAC, 54 (22) TAC + MMF, 52 (21) TAC + mTORi, 12 (5) TAC + steroid, 27 (11) TAC + MMF + steroid, 15 (6) MMF, 24 (10) MMF + mTORi, 10 (4) MMF + steroid, 3 (1) mTORi, 11 (5) mTORi + steroid, 2 (1) Steroid, 2 (1)	Yes	CNIs withdrawal 7% CNIs reduction 16% Antimetabolites withdrawal 14% mTORi withdrawal 4% Other 2%
Bösch et al. [15]	SOT 7; of them, 2 LT	Everolimus + MMF, 1 (50) MMF, 1 (50)	Yes	MMF withdrawal 50%
Choudhury et al. [16]	6	TAC, 2 (33) TAC + MMF, 2 (33) Steroid, 1 (17) Everolimus, 1 (17)	Yes	TAC and MMF withdrawal and start steroids (17) TAC +and MMF withdrawal and start steroids (17) TAC withdrawal and start steroids (33) Several adjustments (17)
Colmenero et al. [17]	111	**Single agent** CNIs, 24 (31) **Combination** CNIs + MMF, 29 (26) CNIs + Everolimus, 9 (8) MMF +/- Everolimus, 37 (33)	NA	NA
Dale et al. [18]	2	TAC + steroid, 1 (50) TAC + MMF + steroid, 1 (50)	Yes	Prednisone reduction 100% (perioperative)
Dhampalwar et al. [19]	12	TAC-based, 10 (83) Cys-based, 1 (8) Everolimus-based, 1 (8) (Not available data on MMF)	Yes	MMF reduction in most patients
Eslami et al. [20]	1	TAC + steroid	No	(perioperative)
Felldin et al. [21]	SOT 53; of them, 8 LT and 3 kidney–LT	TAC, 3 (27) TAC + steroid, 1 (9) TAC + MMF, 2 (18) TAC + MMF + steroid, 3 (27) TAC + AZA, 1 (9) TAC + steroid + MTX, 1 (9)	Yes	MMF withdrawal 27% MMF reduction 9% MTX withdrawal 9% TAC reduction 9%
Fung et al. [22]	1	TAC + MMF	No	-
Hann et al. [24]	3	TAC + AZA + steroid, 3 (100)	NA	NA
Hatami et al. [25]	1	TAC	Yes	TAC withdrawal
Hayashi et al. [26]	1	TAC	No	
Huang et al. [27]	1	TAC + MMF	Yes	Reduction of 50% (drug-drug interaction)
Jamir et al. [28]	1	TAC + MMF + steroid	Yes	MMF withdrawal Steroids i.v.
Kates et al. [29]	SOT 4; of them, 1 LT	Cys	No	
Kolonko et al. [30]	SOT 4; of them, 1 LT	TAC + MMF + steroid	Yes	MMF withdrawal 100%
Liu et al. [31]	1	TAC	Yes	TAC withdrawal Steroids i.v.
Mathiasen et al. [33]	1	TAC + MMF	No	-
Mehta et al. [34]	SOT 11; of them, 3 LT	TAC + steroid, 1 (33) TAC + MMF, 1 (33) TAC + MMF + steroid, 1 (33)	Yes	TAC and MMF withdrawal 33%
Modi et al. [35]	1	TAC + MMF + steroid	Yes	MMF withdrawal TAC reduction
Niknam et al. [36]	2	TAC + MMF + steroid, 2 (100)	Yes	MMF reduction 100%
Nikoupour et al. [37]	1	Tac + MMF + steroid	Yes	MMF reduction
Prieto et al. [38]	1	Basiliximab + MMF + TAC + steroid	Yes	Several adjustments (perioperative)
Qin et al. [39]	1	TAC + steroid	Yes	Several adjustments (perioperative)
Rabiee et al. [40]	112	TAC, 103 (92) MMF, 56 (50) Steroid, 34 (30) Cys, 7 (6) mTOR inhibitors, 4 (4) AZA, 1 (1)	Yes	**Patients with liver injury** (ALT > 2xULN) n = 81 MMF withdrawal 33% TAC reduction 26% TAC withdrawal 5%
Rauber et al. [41]	8	TAC, 1 (13) TAC + MMF, 4 (50) TAC + mTORi, 1 (13) Cys, 1 (13) mTORi, 1 (13)	NA	-
Sessa et al. [42]	1	TAC	No	-
Terrabuio et al. [43]	4	TAC + MMF + steroid, 3 (75) TAC + AZA, 1 (25)	Yes	MMF withdrawal and steroid reduction 50% MMF withdrawal and steroid increase 25% MMF withdrawal and TAC reduction 25%
Waisberg et al. [44]	5	NA for LT	Yes	Reduction 40% Withdrawal 20% Increase 20% (ACR) (all perioperative)
Wei et al. [46]	1	TAC	Yes	TAC withdrawal

Abbreviations: AZA: azathioprine; CNI: calcineurin inhibitors; Cys: cyclosporine; TAC: tacrolimus; MMF: mycophenolate mofetil; mTORi: mammalian target of rapamycin inhibitor; SOT: solid organ transplantation; LT: liver transplant; NA: not applicable; MTX: methotrexate.

**Table 3:** Summary of the included studies concerning immunological response of LT recipients after COVID-19.

First Author	Country	Number of Patients	Type of Test	Type of Assay	Main Conclusions
Zilla et al. [50]	USA	SOT 3; of them, 1 LT and 1 kidney–LT	Anti-SARS-CoV-2 (S1 subunit) IgA and IgG	EUROIMMUN^®^	Delayed serological response and worst outcome
Burack et al. [51]	USA	SOT 70; of them, 14 LT	Anti-SARS-CoV-2 (antinucleocapsid antigen) IgM, IgG, and IgA	Roche Elecsys^®^	80% of liver transplant recipients turned positive
Favà et al. [49]	Spain	SOT 28; of them, 5 LT	Anti-SARS-CoV-2 IgM and IgG + T cell responses	MaglumiTM 2019 (Snibe Diagnostic^®^) + AID^®^ Gmbh	SOT and immunocompetent patients achieved a similarly robust serological and functional T cell immune response, albeit with a certain delay.
Caballeros-Marcos et al. [48]	Spain	71 LT	Anti-SARS-CoV-2 (antinucleocapsid antigen) IgG	Abbott ARCHITECT i2000^®^	LT recipients, compared to immunocompetent patients, showed a lower incidence of antinucleocapsid IgG antibodies at 3 months and at 6 months.

Abbreviations: SOT: solid organ transplantation; LT: liver transplant.

**Table 4:** Summary of the included study concerning vaccination against SARS-CoV-2 response in LT recipients.

First Author	Country	Number of Patients	Type of Vaccine	Type of Assay	Side Effect	Main Conclusions
Boyarsky et al. [54]	USA	SOT 658; of them, 129 LT	mRNA-1273 (Moderna) 307 SOT BNT162b2 (Pfizer-BioNTech) 342 SOT	Anti-SARS-CoV-2 (S1 subunit) IgA and IgG (EUROIMMUN^®^) and Anti-SARS-CoV-2 (antinucleocapsid antigen) IgM, IgG, and IgA (Roche Elecsys^®^)	-	Humoral response to 2 doses of mRNA SARS-CoV-2 vaccine among SOT was present, although participants without a response after dose 1 had generally low antibody levels. Poor humoral response was associated with use of antimetabolite immunosuppression.
Boyarsky et al. [56]	USA	SOT 187; of them, 26 LT	mRNA-1273 (Moderna) 94 SOT BNT162b2 (Pfizer-BioNTech) 93 SOT	-	**Local reactions:** pain (61%) swelling (16%) **Systemic reactions:** fever (4%) chills (9%) fatigue (38%) headache (32%) myalgias (15%)	SOT patients, after 1 dose of vaccine, experienced typically minimal perivaccine reactogenicity similar to reported rates in non-SOT patients.
Mazzola et al. [52]	France	SOT 143; of them, 56 LT	BNT162b2 (Pfizer-BioNTech)	Anti-SARS-CoV-2 (Receptor Binding Domain of S1) IgG (Abbott Alinity i^®^)	**Local reactions:** pain (26%) **Systemic reactions:** fatigue (14%) headache (6%)	Low antibody response 28 days after 2 doses of vaccine among SOT recipients, with 28.6% of seroconversion, particularly for kidney and heart SOT.
Miele et al. [55]	Italy	SOT 16; of them, 4 LT	BNT162b2 (Pfizer-BioNTech)	Anti-SARS-CoV-2 spike protein (S1/S2) (DiaSorin^®^) and ex vivo IFN-γ- ELISpot assay (Mabtech^®^)	-	Humoral and T-cell responses were significantly lower in SOT recipients than in immunocompetent group.
Rabinowich et al. [53]	Israel	80 LT	BNT162b2 (Pfizer-BioNTech)	Anti-SARS-CoV-2 (antinucleocapsid antigen) IgG (Abbott ARCHITECT i2000^®^) and anti-spike protein (S1/S2) (DiaSorin^®^)	-	Positive serology was observed in only 47.5% (*p* < 0.001) of LT recipients. Predictors for negative response among LT recipients were older age, lower estimated glomerular filtration rate, and treatment with high-dose steroids and mycophenolate mofetil.

Abbreviations: SOT: solid organ transplantation; LT: liver transplant.

## Data Availability

Not applicable.

## References

[1] Zhou P., Yang X.-L., Wang X.-G., Hu B., Zhang L., Zhang W., Si H.-R., Zhu Y., Li B., Huang C.-L. (2020). A pneumonia outbreak associated with a new coronavirus of probable bat origin. Nature.

[2] Suleyman G., Fadel R.A., Malette K.M., Hammond C., Abdulla H., Entz A., Demertzis Z., Hanna Z., Failla A., Dagher C. (2020). Clinical Characteristics and Morbidity Associated with Coronavirus Disease 2019 in a Series of Patients in Metropolitan Detroit. JAMA Netw. Open.

[3] Mao L., Jin H., Wang M., Hu Y., Chen S., He Q., Chang J., Hong C., Zhou Y., Wang D. (2020). Neurologic Manifestations of Hospitalized Patients with Coronavirus Disease 2019 in Wuhan, China. JAMA Neurol..

[4] Hu Z., Song C., Xu C., Jin G., Chen Y., Xu X., Ma H., Chen W., Lin Y., Zheng Y. (2020). Clinical characteristics of 24 asymptomatic infections with COVID-19 screened among close contacts in Nanjing, China. Sci. China Life Sci..

[5] Loomba R.S., Aggarwal G., Aggarwal S., Flores S., Villarreal E.G., Farias J.S., Lavie C.J. (2021). Disparities in case frequency and mortality of coronavirus disease 2019 (COVID-19) among various states in the United States. Ann. Med..

[6] Palaiodimos L., Kokkinidis D.G., Li W., Karamanis D., Ognibene J., Arora S., Southern W.N., Mantzoros C.S. (2020). Severe obesity, increasing age and male sex are independently associated with worse in-hospital outcomes, and higher in-hospital mortality, in a cohort of patients with COVID-19 in the Bronx, New York. Metabolism.

[7] https://www.who.int.

[8] Bertolini A., Van De Peppel I.P., Bodewes F.A., Moshage H., Fantin A., Farinati F., Fiorotto R., Jonker J.W., Strazzabosco M., Verkade H.J. (2020). Abnormal Liver Function Tests in Patients with COVID-19: Relevance and Potential Pathogenesis. Hepatology.

[9] Polack F.P., Thomas S.J., Kitchin N., Absalon J., Gurtman A., Lockhart S., Perez J.L., Marc G.P., Moreira E.D., Zerbini C. (2020). Safety and Efficacy of the BNT162b2 mRNA Covid-19 Vaccine. N. Engl. J. Med..

[10] Sadoff J., Le Gars M., Shukarev G., Heerwegh D., Truyers C., de Groot A.M., Stoop J., Tete S., Van Damme W., Leroux-Roels I. (2021). Interim Results of a Phase 1–2a Trial of Ad26.COV2.S Covid-19 Vaccine. N. Engl. J. Med..

[11] Shamseer L., Moher D., Clarke M., Ghersi D., Liberati A., Petticrew M., Shekelle P., Stewart L.A., the PRISMA-P Group (2015). Preferred reporting items for systematic review and meta-analysis protocols (PRISMA-P) 2015: Elaboration and explanation. BMJ.

[12] Alconchel F., Cascales-Campos P.A., Pons J.A., Martínez M., Valiente-Campos J., Gajownik U., Ortiz M.L., Martínez-Alarcón L., Parrilla P., Robles R. (2020). Severe COVID-19 after liver transplantation, surviving the pitfalls of learning on-the-go: Three case reports. World J. Hepatol..

[13] Becchetti C., Zambelli M.F., Pasulo L., Donato M.F., Invernizzi F., Detry O., Dahlqvist G., Ciccarelli O., Morelli M.C., Fraga M. (2020). COVID-19 in an international European liver transplant recipient cohort. Gut.

[14] Belli L.S., Fondevila C., Cortesi P.A., Conti S., Karam V., Adam R., Coilly A., Ericzon B.G., Loinaz C., Cuervas-Mons V. (2021). Protective Role of Tacrolimus, Deleterious Role of Age and Comorbidities in Liver Transplant Recipients with Covid-19: Results From the ELITA/ELTR Multi-center European Study. Gastroenterology.

[15] Bösch F., Börner N., Kemmner S., Lampert C., Jacob S., Koliogiannis D., Stangl M., Michel S., Kneidinger N., Schneider C. (2020). Attenuated early inflammatory response in solid organ recipients with COVID-19. Clin. Transplant..

[16] Choudhury A., Reddy G.S., Venishetty S., Pamecha V., Shasthry S.M., Tomar A., Mitra L.G., Prasad V.S.T., Mathur R.P., Bhattacharya D. (2020). COVID-19 in Liver Transplant Recipients—A Series with Successful Recovery. J. Clin. Transl. Hepatol..

[17] Colmenero J., Rodríguez-Perálvarez M., Salcedo M., Arias-Milla A., Muñoz-Serrano A., Graus J., Nuño J., Gastaca M., Bustamante-Schneider J., Cachero A. (2021). Epidemiological pattern, incidence, and outcomes of COVID-19 in liver transplant patients. J. Hepatol..

[18] Dale M., Sogawa H., Seyedsaadat S.M., Wolf D.C., Bodin R., Partiula B., Nog R., Latifi R., John D., Veillette G. (2021). Successful Management of COVID-19 Infection in 2 Early Post-Liver Transplant Recipients. Transplant. Proc..

[19] Dhampalwar S., Saigal S., Choudhary N., Saraf N., Bhangui P., Rastogi A., Thiagrajan S., Soin A.S. (2020). Outcomes of Coronavirus Disease 2019 in Living Donor Liver Transplant Recipients. Liver Transplant..

[20] Eslami P., Moradi M., Moghadam A.D., Pirsalehi A., Lateef S.A., Hadaegh A., Rezai B., Sadeghi A., Aghdaei H.A., Zali M.R. (2020). Lethal outcome of Covid-19 pneumonia in a new liver recipient with neurological manifestation. Gastroenterol. Hepatol. Bed Bench.

[21] Felldin M., Søfteland J.M., Magnusson J., Ekberg J., Karason K., Schult A., Larsson H., Oltean M., Friman V. (2021). Initial Report From a Swedish High-volume Transplant Center After the First Wave of the COVID-19 Pandemic. Transplantation.

[22] Fung M., Chiu C.Y., DeVoe C., Doernberg S.B., Schwartz B.S., Langelier C., Henrich T.J., Yokoe D., Davis J., Hays S.R. (2020). Clinical outcomes and serologic response in solid organ transplant recipients with COVID-19: A case series from the United States. Arab. Archaeol. Epigr..

[23] Hadi Y.B., Naqvi S.F., Kupec J.T., Sofka S., Sarwari A. (2021). Outcomes of Coronavirus Infectious Disease -19 (COVID-19) in Solid Organ Transplant Recipients: A Propensity-matched Analysis of a Large Research Network. Transplantation.

[24] Hann A.J., Lembach H., McKay S.C., Perrin M., Isaac J., Oo Y.H., Mutimer D., Mirza D.F., Hartog H., Perera T. (2020). Controversies regarding shielding and susceptibility to COVID-19 disease in liver transplant recipients in the United Kingdom. Transpl. Infect. Dis..

[25] Hatami B., Moghadam P.K., Zali M. (2020). Presentation of COVID-19 in a liver transplant recipient. Gastroenterol. Hepatol. Bed Bench.

[26] Hayashi K., Ito Y., Yamane R., Yoshizaki M., Matsushita K., Kajikawa G., Kozawa T., Mizutani T., Shimizu Y., Nagano K. (2021). The case of a liver-transplant recipient with severe acute respiratory syndrome coronavirus 2 infection who had a favorable outcome. Clin. J. Gastroenterol..

[27] Huang J., Zheng K.I., George J., Gao H., Wei R., Yan H., Zheng M. (2020). Fatal outcome in a liver transplant recipient with COVID-19. Arab. Archaeol. Epigr..

[28] Jamir I., Lohia P., Pande R.K., Setia R., Singhal A.K., Chaudhary A.A. (2020). Convalescent plasma therapy and remdesivir duo successfully salvaged an early liver transplant recipient with severe COVID-19 pneumonia. Ann. Hepato-Biliary-Pancreat. Surg..

[29] Kates O.S., Fisher C.E., Stankiewicz-Karita H.C., Shepherd A.K., Church E.C., Kapnadak S.G., Lease E.D., Riedo F.X., Rakita R.M., Limaye A.P. (2020). Earliest cases of coronavirus disease 2019 (COVID-19) identified in solid organ transplant recipients in the United States. Arab. Archaeol. Epigr..

[30] Kolonko A., Dudzicz S., Wiecek A., Król R. (2021). COVID-19 infection in solid organ transplant recipients: A single-center experience with patients immediately after transplantation. Transpl. Infect. Dis..

[31] Liu B., Wang Y., Zhao Y., Shi H., Zeng F., Chen Z. (2020). Successful treatment of severe COVID-19 pneumonia in a liver transplant recipient. Arab. Archaeol. Epigr..

[32] Mansoor E., Perez A., Abou-Saleh M., Sclair S.N., Cohen S., Cooper G.S., Mills A., Schlick K., Khan A. (2021). Clinical Characteristics, Hospitalization, and Mortality Rates of Coronavirus Disease 2019 Among Liver Transplant Patients in the United States: A Multicenter Research Network Study. Gastroenterology.

[33] Mathiasen V.D., Oversoe S.K., Ott P., Jensen-Fangel S., Leth S. (2020). Recovery of Moderate Coronavirus Disease 2019 in a Liver Transplant Recipient on Continued Immunosuppression: A Case Report. Transplant. Proc..

[34] Mehta S.A., Rana M.M., Motter J.D., Small C.B., Pereira M.R., Stosor V., Elias N., Haydel B., Florman S., Odim J. (2021). Incidence and Outcomes of COVID-19 in Kidney and Liver Transplant Recipients With HIV: Report from the National HOPE in Action Consortium. Transplantation.

[35] Modi A.R., Koval C.E., Taege A.J., Esfeh J.M., Eghtesad B., Menon K.N., Quintini C., Miller C. (2020). Coronavirus disease 2019 in an orthotopic liver transplant recipient living with human immunodeficiency virus. Transpl. Infect. Dis..

[36] Niknam R., Malek-Hosseini S.A., Hashemieh S.S., Dehghani M. (2020). COVID-19 in Liver Transplant Patients: Report of 2 Cases and Review of the Literature. Int. Med Case Rep. J..

[37] Nikoupour H., Arasteh P., Gholami S., Nikeghbalian S. (2020). Liver transplantation and COVID-19: A case report and cross comparison between two identical twins with COVID-19. BMC Surg..

[38] Prieto M., Gastaca M., Ruiz P., Ventoso A., Palomares I., Íguez-Álvarez R.J.R., Salvador P., Bustamante J., Valdivieso A.A. (2020). A case of COVID-19 immediately after liver transplantation: Not only bad news. Ann. Hepato-Biliary-Pancreat. Surg..

[39] Qin J., Wang H., Qin X., Zhang P., Zhu L., Cai J., Yuan Y., Li H. (2020). Perioperative Presentation of COVID-19 Disease in a Liver Transplant Recipient. Hepatology.

[40] Rabiee A., Sadowski B., Adeniji N., Perumalswami P.V., Nguyen V., Moghe A., Latt N.L., Kumar S., Aloman C., Catana A.M. (2020). Liver Injury in Liver Transplant Recipients with Coronavirus Disease 2019 (COVID-19): U.S. Multicenter Experience. Hepatology.

[41] Rauber C., Tiwari-Heckler S., Pfeiffenberger J., Mehrabi A., Lund F., Gath P., Mieth M., Merle U., Rupp C. (2021). SARS-CoV-2 Seroprevalence and Clinical Features of COVID-19 in a German Liver Transplant Recipient Cohort: A Prospective Serosurvey Study. Transplant. Proc..

[42] Sessa A., Mazzola A., Lim C., Atif M., Pappatella J., Pourcher V., Scatton O., Conti F. (2020). COVID-19 in a liver transplant recipient: Could iatrogenic immunosuppression have prevented severe pneumonia? A case report. World J. Gastroenterol..

[43] Terrabuio D.R.B., Haddad L., Ducatti L., Gouveia L., Rocha-Santos V., Ferreira R.M.T., Darce G.F., Cardoso A.J.A., Carrilho F.J., Andraus W. (2021). Insights in the approach of long-term liver transplant recipients with COVID-19. Transpl. Infect. Dis..

[44] Waisberg D.R., Abdala E., Nacif L.S., Haddad L.B., Ducatti L., Santos V.R., Gouveia L.N., Lazari C.S., Martino R.B., Pinheiro R.S. (2020). Liver transplant recipients infected with SARS-CoV-2 in the early postoperative period: Lessons from a single center in the epicenter of the pandemic. Transpl. Infect. Dis..

[45] Webb G.J., Marjot T., Cook J.A., Aloman C., Armstrong M.J., Brenner E.J., Catana M.-A., Cargill T., Dhanasekaran R., García-Juárez I. (2020). Outcomes following SARS-CoV-2 infection in liver transplant recipients: An international registry study. Lancet Gastroenterol. Hepatol..

[46] Wei L., Liu B., Zhao Y., Chen Z. (2021). Prolonged shedding of SARS-CoV-2 in an elderly liver transplant patient infected by COVID-19: A case report. Ann. Palliat. Med..

[47] Rodriguez-Peralvarez M., Salcedo M., Colmenero J., Pons J.A. (2021). Modulating immunosuppression in liver transplant patients with COVID-19. Gut.

[48] Caballero-Marcos A., Salcedo M., Alonso-Fernández R., Rodríguez-Perálvarez M., Olmedo M., Morales J.G., Cuervas-Mons V., Cachero A., Loinaz-Segurola C., Iñarrairaegui M. (2021). Changes in humoral immune response after SARS-CoV-2 infection in liver transplant recipients compared to immunocompetent patients. Arab. Archaeol. Epigr..

[49] Favà A., Donadeu L., Sabé N., Pernin V., González-Costello J., Lladó L., Meneghini M., Charmetant X., García-Romero E., Cachero A. (2021). SARS-CoV-2-specific serological and functional T cell immune responses during acute and early COVID-19 convalescence in solid organ transplant patients. Arab. Archaeol. Epigr..

[50] Zilla M.L., Keetch C., Mitchell G., McBreen J., Shurin M.R., E Wheeler S. (2021). SARS-CoV-2 Serologic Immune Response in Exogenously Immunosuppressed Patients. J. Appl. Lab. Med..

[51] Burack D., Pereira M.R., Tsapepas D.S., Harren P., Farr M.A., Arcasoy S., Cohen D.J., Mohan S., Emond J.C., Hod E.A. (2021). Prevalence and predictors of SARS-CoV-2 antibodies among solid organ transplant recipients with confirmed infection. Arab. Archaeol. Epigr..

[52] Mazzola A., Todesco E., Drouin S., Hazan F., Marot S., Thabut D., Varnous S., Soulié C., Barrou B., Marcelin A.-G. (2021). Poor Antibody Response After Two Doses of Severe Acute Respiratory Syndrome Coronavirus 2 (SARS-CoV-2) Vaccine in Transplant Recipients. Clin. Infect. Dis..

[53] Rabinowich L., Grupper A., Baruch R., Ben-Yehoyada M., Halperin T., Turner D., Katchman E., Levi S., Houri I., Lubezky N. (2021). Low immunogenicity to SARS-CoV-2 vaccination among liver transplant recipients. J. Hepatol..

[54] Boyarsky B.J., Werbel W.A., Avery R.K., Tobian A.A.R., Massie A.B., Segev D.L., Garonzik-Wang J.M. (2021). Antibody Response to 2-Dose SARS-CoV-2 mRNA Vaccine Series in Solid Organ Transplant Recipients. JAMA.

[55] Miele M., Busà R., Russelli G., Sorrentino M.C., Di Bella M., Timoneri F., Mularoni A., Panarello G., Vitulo P., Conaldi P.G. (2021). Impaired anti-SARS-CoV-2 humoral and cellular immune response induced by Pfizer-BioNTech BNT162b2 mRNA vaccine in solid organ transplanted patients. Arab. Archaeol. Epigr..

[56] Boyarsky B.J., Ou M.T., Greenberg R.S., Teles A.T., Werbel W.A., Avery R.K., Massie A.B., Segev D.L., Garonzik-Wang J.M. (2021). Safety of the First Dose of SARS-CoV-2 Vaccination in Solid Organ Transplant Recipients. Transplantation.

[57] Li L.-Q., Huang T., Wang Y.-Q., Wang Z.-P., Liang Y., Huang T.-B., Zhang H.-Y., Sun W., Wang Y. (2020). COVID-19 patients’ clinical characteristics, discharge rate, and fatality rate of meta-analysis. J. Med. Virol..

[58] Hussain A., Mahawar K., Xia Z., Yang W., El-Hasani S. (2020). RETRACTED: Obesity and mortality of COVID-19. Meta-analysis. Obes. Res. Clin. Pract..

[59] Kumar A., Arora A., Sharma P., Anikhindi S.A., Bansal N., Singla V., Khare S., Srivastava A. (2020). Is diabetes mellitus associated with mortality and severity of COVID-19? A meta-analysis. Diabetes Metab. Syndr. Clin. Res. Rev..

[60] Li X., Xu S., Yu M., Wang K., Tao Y., Zhou Y., Shi J., Zhou M., Wu B., Yang Z. (2020). Risk factors for severity and mortality in adult COVID-19 inpatients in Wuhan. J. Allergy Clin. Immunol..

[61] Watt K.D., Charlton M.R. (2010). Metabolic syndrome and liver transplantation: A review and guide to management. J. Hepatol..

[62] Becchetti C., Dirchwolf M., Banz V., Dufour J.-F. (2020). Medical management of metabolic and cardiovascular complications after liver transplantation. World J. Gastroenterol..

[63] Tian Y., Rong L., Nian W., He Y. (2020). Review article: Gastrointestinal features in COVID-19 and the possibility of faecal transmission. Aliment. Pharmacol. Ther..

[64] Mao R., Qiu Y., He J.-S., Tan J.-Y., Li X.-H., Liang J., Shen J., Zhu L.-R., Chen Y., Iacucci M. (2020). Manifestations and prognosis of gastrointestinal and liver involvement in patients with COVID-19: A systematic review and meta-analysis. Lancet Gastroenterol. Hepatol..

[65] Zhang H., Kang Z., Gong H., Xu D., Wang J., Li Z., Li Z., Cui X., Xiao J., Zhan J. (2020). Digestive system is a potential route of COVID-19: An analysis of single-cell coexpression pattern of key proteins in viral entry process. Gut.

[66] Xiao F., Tang M., Zheng X., Liu Y., Li X., Shan H. (2020). Evidence for Gastrointestinal Infection of SARS-CoV-2. Gastroenterology.

[67] Perisetti A., Goyal H., Gajendran M., Boregowda U., Mann R., Sharma N. (2020). Prevalence, Mechanisms, and Implications of Gastrointestinal Symptoms in COVID-19. Front. Med..

[68] Bajaj J.S., Kakiyama G., Cox I.J., Nittono H., Takei H., White M., Fagan A., Gavis E.A., Heuman D.M., Gilles H.C. (2018). Alterations in gut microbial function following liver transplant. Liver Transplant..

[69] Sun L., Yang Y.-S., Qu W., Zhu Z.-J., Wei L., Ye Z.-S., Zhang J.-R., Sun X.-Y., Zeng Z.-G. (2017). Gut microbiota of liver transplantation recipients. Sci. Rep..

[70] Jiang J.-W., Ren Z.-G., Lu H.-F., Zhang H., Li A., Cui G.-Y., Jia J.-J., Xie H.-Y., Chen X.-H., He Y. (2018). Optimal immunosuppressor induces stable gut microbiota after liver transplantation. World J. Gastroenterol..

[71] Pan L., Mu M., Yang P., Sun Y., Wang R., Yan J., Li P., Hu B., Wang J., Hu C. (2020). Clinical Characteristics of COVID-19 Patients With Digestive Symptoms in Hubei, China: A Descriptive, Cross-Sectional, Multicenter Study. Am. J. Gastroenterol..

[72] Livanos A.E., Jha D., Cossarini F., Gonzalez-Reiche A.S., Tokuyama M., Aydillo T., Parigi T.L., Ladinsky M.S., Ramos I., Dunleavy K. (2021). Intestinal Host Response to SARS-CoV-2 Infection and COVID-19 Outcomes in Patients with Gastrointestinal Symptoms. Gastroenterology.

[73] Krantz S.G., Rao A.S.S. (2020). Level of underreporting including underdiagnosis before the first peak of COVID-19 in various countries: Preliminary retrospective results based on wavelets and deterministic modeling. Infect. Control. Hosp. Epidemiol..

[74] Fix O.K., Hameed B., Fontana R.J., Kwok R.M., McGuire B.M., Mulligan D.C., Pratt D.S., Russo M.W., Schilsky M.L., Verna E.C. (2020). Clinical Best Practice Advice for Hepatology and Liver Transplant Providers during the COVID-19 Pandemic: AASLD Expert Panel Consensus Statement. Hepatology.

[75] Russell B., Moss C., George G., Santaolalla A., Cope A., Papa S., Van Hemelrijck M. (2020). Associations between immune-suppressive and stimulating drugs and novel COVID-19—A systematic review of current evidence. Ecancermedicalscience.

[76] Allison A.C., Eugui E.M. (2000). Mycophenolate mofetil and its mechanisms of action. Immunopharmacology.

[77] Wang F., Nie J., Wang H., Zhao Q., Xiong Y., Deng L., Song S., Ma Z., Mo P., Zhang Y. (2020). Characteristics of Peripheral Lymphocyte Subset Alteration in COVID-19 Pneumonia. J. Infect. Dis..

[78] Ma-Lauer Y., Zheng Y., Malesevic M., Von Brunn B., Fischer G., Von Brunn A. (2020). Influences of cyclosporin A and non-immunosuppressive derivatives on cellular cyclophilins and viral nucleocapsid protein during human coronavirus 229E replication. Antivir. Res..

[79] Willicombe M., Thomas D., McAdoo S. (2020). COVID-19 and Calcineurin Inhibitors: Should They Get Left Out in the Storm?. J. Am. Soc. Nephrol..

[80] Galipeau Y., Greig M., Liu G., Driedger M., Langlois M.-A. (2020). Humoral Responses and Serological Assays in SARS-CoV-2 Infections. Front. Immunol..

[81] Natori Y., Shiotsuka M., Slomovic J., Hoschler K., Ferreira V., Ashton P., Rotstein C., Lilly L., Schiff J., Singer L. (2017). A Double-Blind, Randomized Trial of High-Dose vs Standard-Dose Influenza Vaccine in Adult Solid-Organ Transplant Recipients. Clin. Infect. Dis..

[82] Kamar N., Abravanel F., Marion O., Couat C., Izopet J., Del Bello A. (2021). Three Doses of an mRNA Covid-19 Vaccine in Solid-Organ Transplant Recipients. N. Engl. J. Med..

